# Tertiary centre study highlights low inpatient deintensification and risks associated with adverse outcomes in frail people with diabetes

**DOI:** 10.1016/j.clinme.2024.100029

**Published:** 2024-02-20

**Authors:** Eka Melson, Mohamed Fazil, Hnin Lwin, Anu Thomas, Ting Fong Yeo, Kevin Thottungal, HayMar Tun, Faseeha Aftab, Meri Davitadze, Alison Gallagher, Samuel Seidu, Kath Higgins

**Affiliations:** a Leicester Royal Infirmary, University Hospitals of Leicester NHS Foundation Trust, LE1 5WW, United Kingdom; b Leicester Diabetes Centre, University of Leicester, LE5 4PW, United Kingdom; c Clinic NeoLab, Tbilisi, Georgia; d Institute of Applied Health Research, University of Birmingham, Birmingham, United Kingdom; e Leicester General Hospital, University Hospitals of Leicester NHS Foundation Trust, LE5 4PW, United Kingdom

**Keywords:** Diabetes, Frailty, Quality improvement, Inpatient hypoglycaemia

## Abstract

**Introduction:**

The community deintensification rates in older people with diabetes are low and hospital admission presents an opportunity for medication review. We audited the inpatient assessment and deintensification rate in people with diabetes and frailty. We also identified factors associated with adverse inpatient outcomes.

**Methods:**

A retrospective review of electronic charts was conducted in all people with diabetes and clinical frailty score ≥6 who were discharged from the medical unit in 2022. Data on demographics, comorbidities and background glucose-lowering medications were collected.

**Results:**

Six-hundred-and-sixty-five people with diabetes and moderate/severe frailty were included in our analysis. For people with no HbA1c in the last six months preceding admission, only 9.0% had it assessed during inpatient. Deintensification rates were 19.1%. Factors that were associated with adverse inpatient outcomes included inpatient hypoglycaemia, non-White ethnicity, and being overtreated (HbA1c <7.0% [53 mmol/mol] with any glucose-lowering medication).

**Conclusion:**

The assessment and deintensification rate in secondary care for people with diabetes and frailty is low. Inpatient hypoglycaemia, non-White ethnicity, and overtreatment are important factors in determining inpatient outcomes highlighting the importance of deintensification and the need for an evidence-based risk stratification tool.

**Summary**

**What is already known?**

• People with diabetes and frailty require less intensive treatment due to the risks of hypoglycaemia, falls, and hospital admission.
• Treatment inertia represents a significant issue in this cohort of population with studies showing low rates of community deintensification for people with diabetes and frailty.

**What are the questions?**

• What is the quality of inpatient assessment and management of people with diabetes and frailty?
• What are the factors that are associated with adverse inpatient outcomes in people with diabetes and frailty?

**What was found?**

• This study has shown suboptimal inpatient assessment and low deintensification rates in people with diabetes and moderate/severe frailty.
• Inpatient hypoglycaemia and non-White ethnicity are associated with increased adverse inpatient outcomes.

**What are the implications for practice now?**

• Interventions to improve inpatient assessments of people with diabetes and moderate/severe frailty are needed to improve the overall management of people with diabetes and moderate/severe frailty.
• An evidenced-based stratification tool to identify patients at the highest risk of hypoglycaemia and overtreatment is urgently needed to improve patient care and decrease inpatient morbidity and mortality.

Alt-text: Unlabelled box

## Introduction

Approximately 40% of adults older than 65 years have diabetes and managing diabetes in this age group comes with distinct challenges.^1^ Increasing evidence has shown that the degree of frailty, rather than age, is a better prognostic marker for people with diabetes.^2^^,^^3^ Assessment of frailty is recommended in people with diabetes and is being increasingly adopted in routine clinical practice, with several validated tools recommended by the Joint British Diabetes Societies (JBDS) (Table 1) including the clinical frailty scale (CFS).^4^ When interpreting the CFS, it is crucial to understand that not all older adults are frail as frailty is a complex and dynamic condition that can occur at any age.^5^ Achieving tight glycaemic control can delay the long-term macro- and microvascular complications of diabetes, but it is important to strike a balance between potential benefits and associated risks. Tight glycaemic control in adults with frailty can be controversial due to increased risks of falls, hypoglycaemia, emergency department visits, hospitalisation, and mortality. Decisions regarding the deintensification of glucose-lowering medications in frail populations should be individualised and based on a thorough assessment of the patient's overall health, frailty status, life expectancy, and quality of life goals.^2^^,^^3^

**Table 1:** Tools to detect frailty in people with diabetes as recommended by the Joint British Diabetes Societies (JBDS). Adapted from the JBDS Inpatient care of frail older adults with diabetes.^11^

Assessment tool	
Fried score^21^	Consists of five measures: i. Shrinking: Weight loss (unintentional), sarcopenia (loss of muscle mass. Defined as > 10lbs weight loss in the prior year. ii. Weakness: defined as grip strength in the lowest 20% by gender and body mass index. iii. Self-reported poor endurance or exhaustion iv. Slowness: walking time/15 feet, slowest 20% by gender and body mass index v. Calories per week < 383 in men and < 270 in women ≥ 3 positive criteria: frail phenotype 1 or 2 positive phenotypes: Intermediate or pre-frail
Clinical frailty score (CFS)^4^	Nine-point visual descriptions of physical functioning and care needs that are easy to implement in routine clinical practice. CFS 5 = Mild frailty: people with evidence of slowing and need help with high order instrumental activities of daily living CFS 6 = Moderate frailty: people who need help with all outside activities and with keeping the house. Problems with stairs, bathing and minimal assistant with dressing CFS 7 = Severe frailty: completely dependent for personal care CFS 8 = Very severe frailty: completely dependent for personal care; approaching end of life CFS 9 = Terminally ill: life expectancy of < 6months, who are not otherwise living with severe frailty
FRAIL score^22^	Five components including: (0 = Best; 5 = Worst) i. Fatigue ii. Resistance iii. Ambulation iv. Illness v. Loss of weight Score 3–5 = frailty Score 1–2 = pre-frailty 0 = robust health status
PRISMA 7 questionnaire^23^	Seven simple questions: i. > 85 years old? ii. Male? iii. Any problems that limit activities? iv. Require help on a regular basis? v. Any health problems that require person to stay at home? vi. If need help, can you count on somebody close? vii. Regularly uses stick, walker or wheelchair to move about? More than 3 “Yes” – increased risk for frailty and require further clinical review

Treatment inertia is defined as the failure of healthcare professionals to intensify or deintensify treatment when it is indicated.^6^ The importance of deintensification in people with diabetes and frailty has been increasingly highlighted due to increasing polypharmacy, endocrine deficits, suboptimal food and drink intake, cognitive impairment, cardiovascular disease, and renal dysfunction, that increase susceptibility to hypoglycaemia and its potentially severe consequences.^7^ Moreover, whilst there is good evidence that tight glycaemic control can delay diabetes complications, evidence in people with diabetes and frailty is lacking. Studies on treatment deintensification, despite being limited to the primary care and older people with diabetes (rather than frailty), have shown low rates of deintensification.^8^, ^9^, ^10^ The evidence regarding deintensification in people with diabetes and frailty beyond primary care is, however, still lacking. Factors associated with adverse inpatient outcomes also need further investigation.

The JBDS guideline on inpatient care of frail older adults with diabetes recommends that attempts should be made to access previous glycated haemoglobin (HbA1c) readings carried out in the last 6–12 months.^11^ This will help identify those with the highest risk of inpatient hypoglycaemia—people with an HbA1c of less than 7% (53 mmol/mol).^12^ If no recent HbA1c readings are available, the guideline recommends carrying these out during the admission. As admission to the hospital represents an opportunity for medication review in people with diabetes and frailty, assessing HbA1c upon admission could improve the rate of deintensification and avoid treatment inertia in this group of patients.

The aims of this audit were (i) to identify the proportion of patients who had their HbA1c assessed during their inpatient stay if they had not been carried out six months prior to the admission, (ii) to assess the rates of inpatient deintensification including those with a higher risk of hypoglycaemia, and (iii) to describe the characteristics and outcomes of people with diabetes and moderate/severe frailty admitted to the hospital.

## Methods

### Study population and data source

**Fig. 1:**
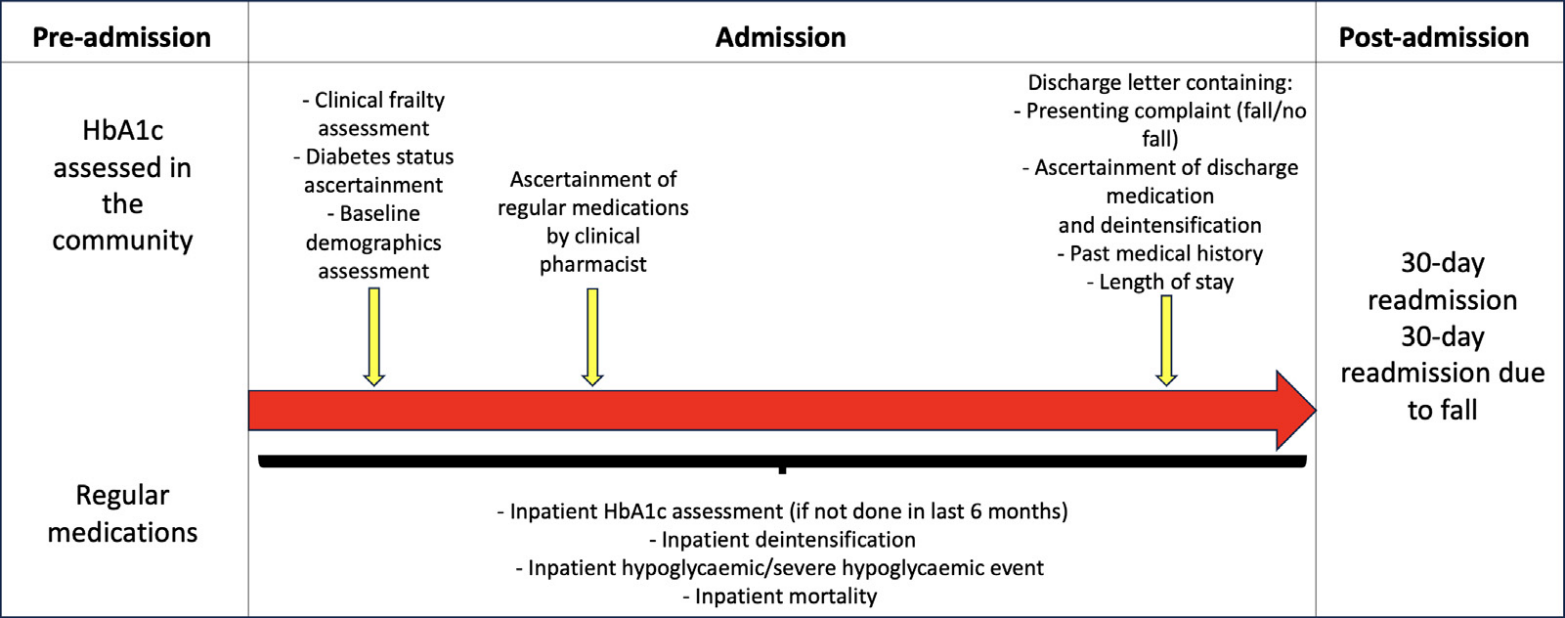
Retrospective data collection for people admitted with diabetes and moderate/severe frailty.

All patients who were admitted to the University Hospitals of Leicester NHS Trust underwent diabetes assessment by frontline clinical staff who ascertained patients’ diabetes status and type of diabetes either by asking the patients and/or looking at the patient's electronic record system. CFS was also calculated for every patient on admission and these data were incorporated into the patients’ electronic chart. Admission medications were ascertained by clinical pharmacists by asking the patients, carers, and/or by accessing their primary care electronic medical records (Fig. 1).

Retrospective electronic charts were reviewed in all patients with diabetes and CFS ≥6 who were discharged from the medical unit at the Leicester Royal Infirmary, University Hospitals of Leicester NHS Trust. Patients were included if they had CFS ≥6 and a history of diabetes ascertained by their past medical history. Data on age, sex, ethnicity, CFS, weight, height, body mass index (BMI, calculated as weight in kilogrammes divided by height in square metres), diabetes-related complications [retinopathy, chronic kidney disease/nephropathy, peripheral arterial disease/amputation, neuropathy, heart failure (heart failure with preserved ejection fraction, heart failure with reduced ejection fraction and congestive heart failure), ischaemic heart disease (stable or unstable angina, previous myocardial infarctions), cerebrovascular disease (previous transient ischaemic attack or previous stroke), hypertension, cognitive impairment/dementia, and dyslipidaemia], and background glucose-lowering medications were collected. Levels of HbA1c on admission, defined as HbA1c levels that were checked prior to admission, and the dates of measurement were also collected. The dates of HbA1c measurement were then used to assess if the HbA1c levels were checked six months prior to the admission date. If the patient was on metformin, information regarding vitamin B12 measurement on admission including the date assessed was collected. Following discharge, the discharge letter and discharge medications were reviewed and compared to the admission medications to assess for any deintensification during their inpatient stay. Data regarding patients’ presenting complaints were checked to ascertain if this episode of admission was due to a fall. Data on inpatient hypoglycaemia, length of stay and 30-day readmission rates were also collected.

This study received registration and approval as an audit and quality improvement programme (QIP) at the University Hospitals of Leicester (QIP reference number: 12278). The UHL QIP committee determined that further ethical approval was not needed since this study pertains to patient improvement within our institution.

### Definition of patient parameters and outcomes

Overtreatment was defined as people with admission HbA1c of <7% (53 mmol/mol) with at least one glucose-lowering medication. Other overtreatment groups were also created with different HbA1c thresholds [<7.5% (59 mmol/mol), <8.0% (64 mmol/mol), and <8.5% (69 mmol/mol)]. These HbA1c levels were chosen due to the heterogeneity of the definitions of overtreatment in older people with diabetes. Any inpatient episode of hypoglycaemia was defined as any capillary blood glucose reading of <4.0 mmol/L during their inpatient stay, whereas severe hypoglycaemia was defined as a capillary blood glucose of <3.0 mmol/L. Inpatient deintensification was defined as a decrease or discontinuation of any glucose-lowering medication without adding another drug, or a reduction in the total daily dose of insulin or a sulphonylurea with or without adding a drug with a lower risk of hypoglycaemia. The change of insulin regimen to insulin with a better hypoglycaemic profile was also considered deintensification. Ethnicity status was categorised into White and non-White (Black – African, Black – Caribbean, Asian – Indian, Asian – Bangladesh, Asian – Pakistan, Asian – Chinese, Asian – Others) due to the small sample of individuals of non-White ethnicities.

### Statistical analysis

Shapiro-Wilk test was used to check for the distribution of the continuous variables. The median and interquartile range (IQR) are presented due to the variables’ non-normality. Descriptive statistics are presented for the categorial variables and are presented as absolute numbers and proportions. Chi-squared test and Mann-Whitney test were conducted to assess the differences between groups for categorical and continuous data, respectively. Multivariate logistic regression was used to assess for any associations of patient factors with inpatient hypoglycaemia, deintensification, and inpatient mortality. Odds ratios are presented with a 95% confidence interval (CI) and models are adjusted for age, sex, ethnicity, CFS, background co-morbidities, background medications, admission HbA1c, and reason for admission. Simple linear regression was used to assess for any association between patient factors and length of stay. Final multivariate linear regression models included age, CFS, BMI, and insulin use. The statistical significance threshold was 2-sided p=0.05. Data were analysed using Stata/SE 17.0 for Mac (Texas, US).

## Results

### Baseline characteristics and rates of overtreatment

A total of 665 people with diabetes and CFS ≥6 was included in our analysis. Median age was 79 years (71–86 years). The majority of people were White (75.8%, n=504/665) with a median CFS of 6 (6-7). 96.4% (n=641/665) of people had type 2 diabetes. The admission medications and baseline co-morbidities are described in Table 2. 27.1% of the people were admitted due to falls. The median length of stay was 8 days (3-16 days). The rate of inpatient hypoglycaemia and inpatient mortality was 18.2% (n=116/637) and 7.4% (n=49/665), respectively. 24.1% (n=153/634) of people were readmitted in a month and of these, 19% (n=29/153) were readmitted due to fall. Appendices 1–4 illustrate the baseline characteristics of people with diabetes and moderate/severe frailty stratified by admission HbA1c, age, sex, and ethnicity, respectively.

**Table 2:** Baseline characteristics of inpatients with diabetes and moderate/severe frailty stratified by HbA1c status.

	Total	Overtreated	Not overtreated (HbA1c ≥7% or 53mmol/mol)	p-value
n (%)	665 (100%)	335/665 (50.4%)	330/665 (49.6%)	
Age [median (IQR)]	79 (71–86)	80 (71–86)	79 (71–85)	0.151
Female, n (%)	345 (51.9%)	180/345 (48.4%)	165/345 (47.8%)	0.336
BMI [median (IQR)]	25.79 (22.1–30.8)	320, 25.01 (21.2–29.9)	316, 26.45 (22.8–31.9)	0.021
**Ethnicity**, n (%)				
White	504/665 (75.8%)	249/335 (74.3%)	255/330 (77.3%)	0.375
Non-white	161/665 (24.2%)	86/335 (25.7%)	75/330 (22.7%)	
**CFS**, n (%)				
Moderate frailty	448/665 (67.4%)	218/335 (65.1%)	230/330 (51.3%)	0.140
Severe frailty	176/665 (26.5%)	96/335 (28.7%)	80/330 (45.5%)	
Very severe frailty	29/665 (4.4%)	12/335 (3.6%)	17/330 (5.2%)	
Terminally ill	12/665 (1.8%)	9/335 (2.7%)	3/330 (0.9%)	
**Diabetes type**, n (%)				
Type 1 diabetes	18/665 (2.7%)	3/18 (16.7%)	15/18 (83.3%)	0.002
Type 2 diabetes	641/665 (96.4%)	327/641 (51.0%)	314/641 (49.0%)	
**Co-morbidities,** n (%)				
Retinopathy	62/665 (9.3%)	22/335 (6.6%)	40/330 (12.1%)	0.014
CKD/Nephropathy	362/665 (54.4%)	180/335 (53.7%)	182/330 (55.2%)	0.713
PAD/Amputation	40/665 (6.0%)	17/335 (5.1%)	23/330 (7.0%)	0.304
Neuropathy	26/665 (3.9%)	11/335 (3.3%)	15/330 (4.5%)	0.401
Heart failure	167/665 (25.1%)	87/335 (26.0%)	80/330 (24.2%)	0.607
Ischaemic heart disease	177/665 (26.6%)	80/335 (23.9%)	97/330 (29.4%)	0.108
Cerebrovascular event	135/665 (20.3%)	65/335 (19.4%)	70/330 (21.2%)	0.562
Hypertension	448/665 (67.4%)	233/335 (69.6%)	215/330 (65.2%)	0.226
Dementia	159/665 (23.9%)	74/335 (22.1%)	85/330 (25.8%)	0.268
Dyslipidaemia	128/665 (19.2%)	55/335 (16.4%)	73/330 (22.1%)	0.062
**Glucose-lowering agents**, n (%)				
Metformin	247/665 (37.1%)	106/335 (31.6%)	141/330 (42.7%)	0.003
Sulphonylureas	60/665 (9.0%)	16/335 (4.8%)	44/330 (13.3%)	0.000
Insulin	222/665 (33.4%)	45/335 (13.4%)	177/330 (53.6%)	0.000
GLP-1 receptor agonist	12/665 (1.8%)	4/335 (1.2%)	8/330 (2.4%)	0.233
DPP4i	119/665 (17.9%)	45/335 (13.4%)	74/330 (22.4%)	0.002
SGLT2i	40/665 (6.0%)	18/335 (5.4%)	22/330 (6.7%)	0.483
At least one glucose-lowering agent, n (%)	467/665 (70.2%)	177/335 (52.8%)	290/330 (87.9%)	<0.001
**Sulphonylureas or Insulin**, n (%)				
None	397/665 (59.7%)	276/335 (82.4%)	121/330 (36.7%)	
At least one	254/665 (38.2%)	57/335 (17.0%)	197/330 (59.7%)	
Both	14/665 (2.1%)	2/335 (0.6%)	12/330 (3.6%)	<0.001
Admission HbA1c [median (IQR)]	6.9% (6.2–8.1) or 52mmol/mol (44–65)	6.2% (5.8–6.6) or 44mmol/mol (40–49)	8.1 (7.5–9.4) or 65mmol/mol (59–79)	<0.001
If on metformin, vitamin B12 checked in the last 1 year, n (%)	136/247 (55.1%)	63/335 (59.4%)	73/330 (51.8%)	0.231
Admission due to fall, n (%)	170/628 (27.1%)	78/335 (24.8%)	92/330 (29.3%)	0.209
Inpatient hypoglycaemia, n (%)	116/637 (18.2%)	45/335 (14.1%)	71/330 (22.3%)	0.007
Inpatient mortality, n (%)	49/665 (7.4%)	29/335 (8.7%)	20/330 (6.1%)	0.200
Length of stay [median (IQR)]	8 (3-16)	8 (3–14.5)	7 (3–16.3)	0.736
Deintensification, n (%)	119/624 (19.1%)	45/335 (14.4%)	74/330 (23.7%)	0.003
One-month readmission, n (%)	153/634 (24.1%)	77/335 (24.2%)	76/330 (24.1%)	0.962
One-month readmission due to fall, n (%)	29/153 (19.0%)	17/335 (22.4%)	12/330 (15.6%)	0.284

IQR = interquartile range; BMI = body mass index; HbA1c = glycated haemoglobin A1c; CFS: clinical frailty score; CKD = chronic kidney disease; PAD = peripheral arterial disease; DPP4i = dipeptidyl peptidase-4 inhibitor; SGLT2i = Sodium-glucose co-transporter-2; Overtreated = HbA1c <7.0% + at least one glucose-lowering medication.

50.4% (n=335/665) of the people with diabetes and frailty were overtreated (HbA1c <7% [53 mmol/mol]) (Table 2, Appendix 1). BMI was slightly higher in people who were not overtreated compared to the overtreated (26.5 kg/m^2^ vs 25.0 kg/m^2^, n=0.021). People who were overtreated were less likely to have retinopathy and be on medications such as metformin, sulphonylureas, insulin, and DPP4i. People who were overtreated had fewer episodes of inpatient hypoglycaemia compared to those who were not overtreated. There were no statistically significant differences in other inpatient outcomes (Table 2).

### Audit of inpatient assessments and rates of deintensification

**Fig. 2:**
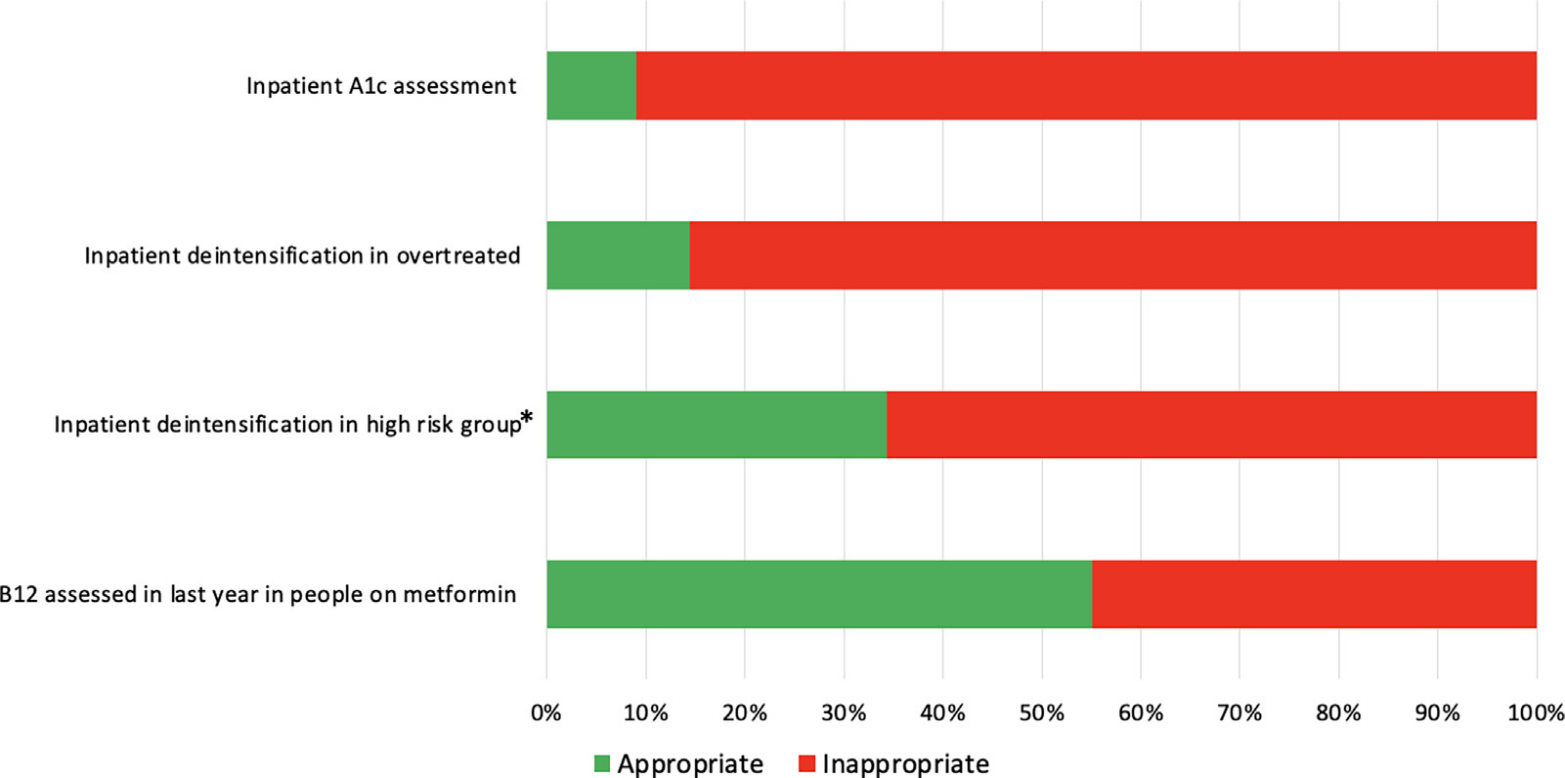
Inpatient assessment and management of people with diabetes and moderate/severe frailty. *people with diabetes and frailty with HbA1c < 7% (53mmol/mol) who are on insulin/sulphonlyurea and/or inpatient hypoglycaemia.

68.1% (n=453/665) of people had their HbA1c assessed 6 months prior to the admission. Of those who did not have their HbA1c checked in the preceding 6 months, only 9.0% (n=19/212) had it checked during the admission. In people who were on metformin, only 55.1% (n=136/247) had their vitamin B12 levels checked in the last 12 months. The rate of deintensification was 19.1% (n=119/625); in the overtreated group deintensification rate was lower, compared to the not overtreated group (14.4% vs 23.7%, p=0.003). Of those who were on insulin and/or sulphonylurea, HbA1c <7.0% (53 mmol/mol) and/or hypoglycaemia, only 34.3% (n=74/216) were deintensified (Fig. 2).

### Factors associated with inpatient hypoglycaemia, deintensification, length of stay, inpatient mortality, and readmission

Factors that were associated with inpatient hypoglycaemia include insulin therapy (OR: 4.74, 95% CI: 3.09–7.27) and a history of retinopathy (OR: 2.06, 95% CI: 1.14–3.72). After adjusting for background factors, insulin therapy (aOR: 5.21, 95% CI: 3.02–8.99) and non-White ethnicity (aOR: 1.70, 95% CI: 1.01–2.88) were associated with higher odds of inpatient hypoglycaemia (Table 3). Insulin therapy increased the odds of severe hypoglycaemia by threefold (aOR: 3.39, n=0.005), compared to those without insulin therapy. Interestingly, metformin was a protective factor (aOR: 0.32, 95% CI: 0.12–0.82) for inpatient severe hypoglycaemia, compared to those without metformin (Appendix 5). People with inpatient hypoglycaemia and severe hypoglycaemia were independently associated with being deintensified compared to those without hypoglycaemia and severe hypoglycaemia, respectively (Appendix 6). Both inpatient hypoglycaemia and severe hypoglycaemia were associated with increased length of stay adjusted for insulin use, age, CFS, and BMI (Table 4, Appendix 7).

**Table 3:** Unadjusted and adjusted odds ratio for developing inpatient hypoglycaemia in people with diabetes and moderate/severe frailty.

Variable	OR (95% CI)	p-value	aOR (95% CI)	p-value
Age > 65	0.92 (0.51 - 1.65)	0.782	1.45 (0.70 - 3.01)	0.426
Female sex	0.99 (0.66 - 1.48)	0.97	0.94 (0.59 - 1.49)	0.793
Ethnic minority	1.54 (0.98 - 2.41)	0.063	1.70 (1.01 - 2.88)	0.049
**CFS group**				
Moderate Frailty	1		1	
Severe Frailty	0.97 (0.61 - 1.54)	0.903	0.92 (0.54 - 1.56)	0.752
Very Severe Frailty	0.81 (0.27 - 2.41)	0.700	0.82 (0.25 - 2.71)	0.748
Terminally ill	1.11 (0.23 - 5.32)	0.897	1.32 (0.22 - 7.69)	0.757
Retinopathy	2.06 (1.14 - 3.72)	0.017	1.56 (0.78 - 3.10)	0.209
Chronic kidney disease/Nephropathy	1.00 (0.67 - 1.50)	0.997	0.91 (0.56 - 1.50)	0.725
Peripheral arterial disease/Amputation	1.19 (0.53 - 2.66)	0.671	0.79 (0.32 - 1.98)	0.617
Neuropathy	1.38 (0.54 - 3.50)	0.504	0.95 (0.32 - 2.79)	0.925
Heart Failure	1.17 (0.74 - 1.84)	0.501	1.09 (0.64 - 1.88)	0.747
Ischaemic heart disease	1.04 (0.67 - 1.65)	0.853	0.97 (0.57 - 1.63)	0.905
Cerebrovascular events	0.76 (0.45 - 1.29)	0.306	0.64 (0.36 - 1.16)	0.143
Hypertension	1.00 (0.65 - 1.54)	0.99	1.00 (0.60 - 1.66)	0.998
Dementia	0.88 (0.54 - 1.42)	0.59	0.81 (0.47 - 1.39)	0.448
Dyslipidaemia	0.77 (0.45 - 1.32)	0.347	0.69 (0.38 - 1.27)	0.235
Metformin	0.78 (0.50 - 1.19)	0.246	0.89 (0.53 - 1.47)	0.641
Sulphonylureas	1.55 (0.81 - 2.94)	0.182	2.02 (0.93 - 4.38)	0.075
Insulin	4.74 (3.09 - 7.27)	< 0.01	5.21 (3.02 - 8.99)	<0.001
DPP4i	1.06 (0.63 - 1.78)	0.823	0.92 (0.51 - 1.65)	0.767
SGLT2i	0.66 (0.25 - 1.72)	0.39	0.30 (0.08 - 1.06)	0.061
Admission HbA1c <7% or 53 mmol/mol	0.57 (0.38 - 0.86)	0.007	1.15 (0.68 - 1.95)	0.591
Overtreated 1	1.14 (0.73 - 1.78)	0.567	1.41 (0.84 - 2.38)	0.194
Overtreated 2	1.12 (0.74 - 1.70)	0.586	1.35 (0.83 - 2.22)	0.223
Overtreated 3	1.20 (0.80 - 1.80)	0.377	1.32 (0.82 - 2.13)	0.251
Overtreated 4	1.20 (0.80 - 1.80)	0.371	1.21 (0.75 - 1.98)	0.423
Admission due to fall	1.09 (0.70 - 1.72)	0.697	1.22 (0.72 - 2.06)	0.455

95% CI = confidence interval; OR = odds ratio; aOR = adjusted for age, sex, ethnicity, CFS, background co-morbidities, background medications, admission HbA1c and admission due to fall; DPP4i = dipeptidyl peptidase-4 inhibitor; SGLT2i = Sodium-glucose co-transporter-2.Overtreated 1 = HbA1c <7.0% or 53mmol/mol + at least one glucose-lowering medication; Overtreated 2 = HbA1c <7.5% or 59 mmol/mol+ at least one glucose-lowering medication; Overtreated 3 = HbA1c <8.0% or 64 mmol/mol + at least one glucose-lowering medication; Overtreated 4 = HbA1c <8.5% or 69 mmol/mol + at least one glucose-lowering medication.

**Table 4:** Multiple linear regression model showing association between inpatient hypoglycaemia and length of stay.

Variable	Regression coefficient	95% CI	p-value
Inpatient hypoglycaemia	6.26	3.27 - 9.24	<0.001
Insulin	1.19	-1.33 - 3.70	0.354
Age	0.02	-0.09 - 0.13	0.711
CFS	-0.61	-2.36 - 1.15	0.498
BMI	0.13	0.02 - 0.27	0.091
Constant	9.79	-6.11 - 25.7	0.227
R^2^ = 0.0348			
Multiple linear regression model showing an association between inpatient severe hypoglycaemia and length of stay.			
Inpatient severe hypoglycaemia	8.17	3.49 - 12.8	0.001
Insulin	2.11	-0.33 - 4.56	0.09
Age	0.03	-0.08 - 0.14	0.637
CFS	-0.63	-2.40 - 1.13	0.48
BMI	0.14	-0.01 - 0.29	0.063
Constant	9.45	-6.69 - 25.6	0.251
R^2^ = 0.027			

95% CI = confidence interval; CFS = clinical frailty score; BMI = body mass index

Adjusting for background factors, being overtreated (HbA1c <7% [53 mmol/mol] with at least one glucose-lowering medication) was associated with increased odds of inpatient mortality (aOR: 3.83, 95% CI: 1.06–13.8), compared to those that were not overtreated (HbA1c ≥7% [53 mmol/mol]). People with inpatient hypoglycaemia also had higher odds of inpatient mortality compared to those without inpatient hypoglycaemia (aOR: 3.57, 95% CI: 1.02–12.4). Those with hypertension had lower odds of inpatient mortality, compared to those without hypertension (aOR: 0.15, 95% CI: 0.04–0.50). Non-White ethnicity had over two-fold increased odds of inpatient mortality, compared to White ethnicity (OR: 2.11, 95% CI: 1.16–3.87). However, this association was lost after adjusting for inpatient hypoglycaemia (aOR: 1.41, 95% CI: 0.37–5.39) (Appendix 8).

After adjusting for other variables, ischaemic heart disease was associated with higher odds of readmission to the hospital in a month (aOR: 1.67, 95% CI: 1.07–2.61). People who were aged ≥65 years were less likely to be readmitted, compared to those aged <65 years (aOR: 0.46, 95% CI: 0.25–0.85) (Appendix 9). As for the readmissions, those with initial admission due to a fall were more likely to be readmitted in a month due to a fall (aOR: 4.02, 95% CI: 1.04–15.5). Metformin appeared to decrease the odds of one-month readmission due to a fall (aOR: 0.22, 95% CI: 0.05–0.92) (Appendix 10).

## Discussion

Our results show suboptimal assessment of HbA1c in people with diabetes and moderate/severe frailty admitted to the hospital. The rate of deintensification in people with a high risk of falls and hypoglycaemia was also low, suggesting the treatment inertia in this population. Additionally, the inpatient hypoglycaemia rate was unacceptably high, possibly due to pre-admission treatment inertia, and correlated with insulin therapy and non-White ethnicity. Inpatient hypoglycaemic episodes were also associated with a prolonged stay. Factors associated with inpatient mortality included being overtreated and having an episode of inpatient hypoglycaemia.

Regrettably, our audit has shown that the JBDS recommendation for HbA1c measurements^11^ in people with diabetes and frailty is not part of routine clinical practice, causing an incomplete assessment of older people with diabetes and frailty. Furthermore, the absence of HbA1c means missed chances for deintensification during hospitalisation, perpetuating therapeutic inertia. As part of the inpatient assessment of people with diabetes and moderate/severe frailty, identifying those with the highest risk of morbidity and mortality from overtreatment is also crucial. Considering the importance of individualised care in the older population, several guidelines for HbA1c targets in older people with diabetes have been published. Depending on the recommendations, the definition of overtreatment varies by HbA1c ranging from <7.0% to <8.5% (53–69 mmol/mol) with or without glucose-lowering medication.^13^ Our chosen target of HbA1c <7.0% (53 mmol/mol) with at least one glucose-lowering medication was conservative as it allowed us to identify the lowest rate of deintensification in these patients upon admission to the hospital. Furthermore, considering the different definitions used for overtreatment in our study, the HbA1c threshold of <7.0% (53 mmol/mol) seems to be the only group that was independently associated with higher odds of inpatient mortality. However, it is important to note that HbA1c levels may be inaccurate in conditions such as anaemia and iron deficiency hence reliance on HbA1c may not be appropriate in all patients.^14^

Our findings show that only 14.4% of overtreated people with diabetes and moderate/severe frailty were deintensified during admission. This could be due to the low rate of HbA1c assessment and other confounding factors such as stress hyperglycaemia or patient preferences that prevented deintensification. In people who were on insulin and/or sulphonylurea with HbA1c <7.0% (53 mmol/mol) and/or inpatient hypoglycaemia, the deintensification rate was slightly higher at 34.3%. No immediate benefit was seen with inpatient deintensification such as a reduced one-month readmission rate or reduced readmission due to falls. Observing such benefits is probably premature at this duration and the low sample size of readmitted people may also play a role. Regardless, reducing polypharmacy has been shown to benefit people who are frail independent of HbA1c levels.^10^ The evidence for the benefits of deintensification in older people with diabetes and frailty is also emerging with studies suggesting the benefits of deintensification outweigh the harm regardless of the co-morbidities.^7^^,^^15^ Moreover, episodes of hypoglycaemia in older people with diabetes and frailty are associated with increased mortality and morbidity including increased cardiovascular risks further highlighting the need for deintensification especially for those with the highest risk.^16^^,^^17^ In line with this, our findings also showed that people with inpatient hypoglycaemia who were overtreated had higher odds of inpatient mortality.

**Fig. 3:**
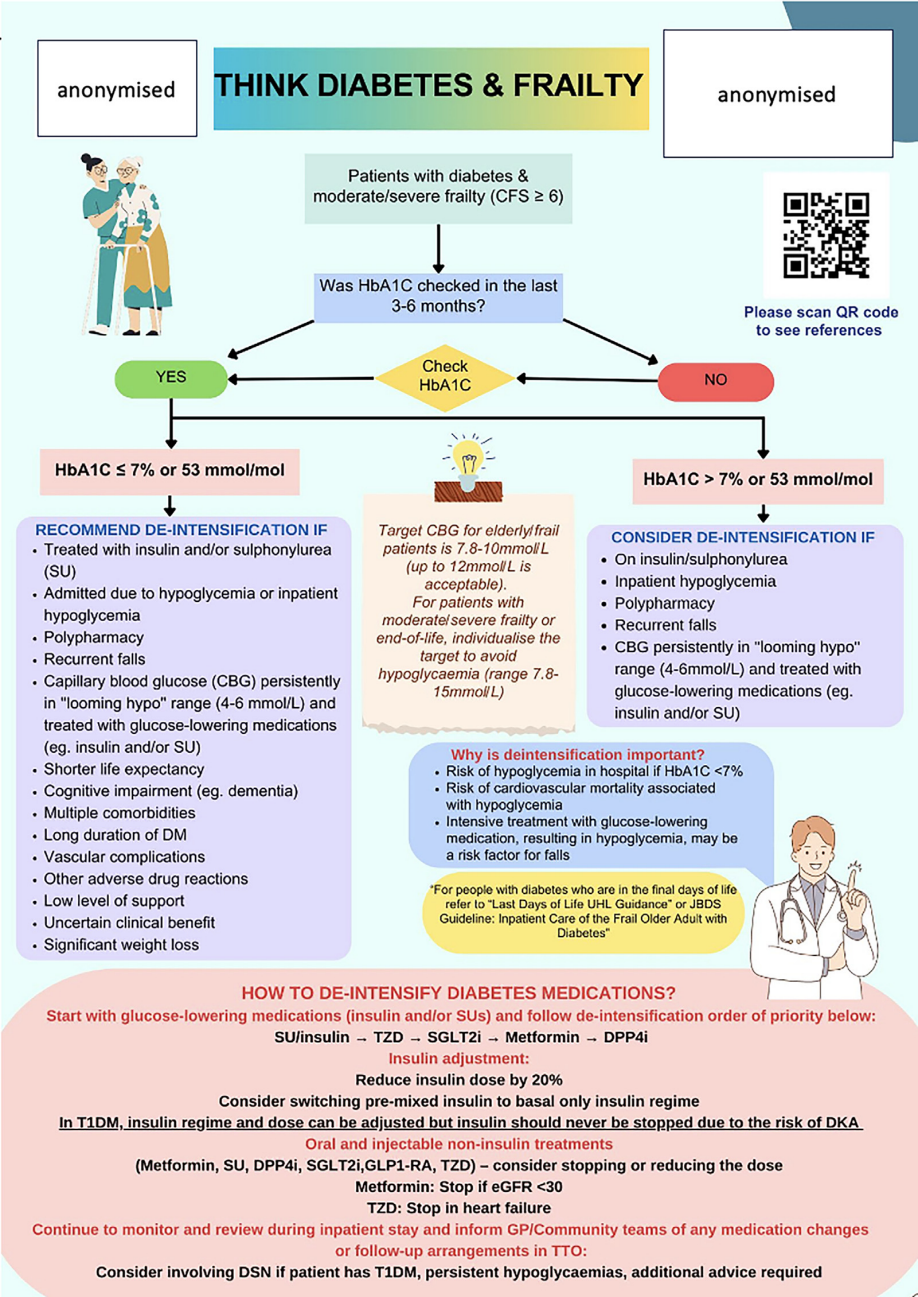
Inpatient guidance for assessment and deintensification in people with diabetes and frailty.

On the other hand, several studies have been conducted to assess the rates of deintensification in older people with diabetes. However, most of them did not consider frailty status and were conducted in primary care setting.^8^^,^^9^ In an Australian study of older people with type 2 diabetes who presented to the hospital with severe hypoglycaemia, the deintensification rate was found to be less than 50% within 100 days of presentation.^10^ People with frailty were more likely to be deintensified compared to those without. However, the study was only limited to people older than 65 years hence omitting younger people with frailty.^10^ In contrast, our study provided new evidence in younger people with diabetes and moderate/severe frailty with only 19.7% of deintensification in this population, similar to older people. Evidence-based interventions and guidance for inpatient assessment and deintensification of people with diabetes and moderate/severe frailty are needed to improve the care of such patients and reduce the burden of primary care. Our institution is developing a quality improvement project to guide the assessment and deintensification for frail people with diabetes (Fig. 3). This responsibility falls to the managing physicians, but the diabetes inpatient team (DIT) plays a crucial role in identifying high-risk patients and assisting with complex cases, particularly those involving multiple treatments or insulin regime adjustment that might be indicated.

In our study, admission HbA1c and being overtreated were not associated with inpatient hypoglycaemia which could be explained by the low proportion of people being on agents with a higher risk of hypoglycaemia. Inpatient hypoglycaemia appears to be one of the most important factors that impact inpatient outcomes in people with diabetes and frailty. In our study, having inpatient hypoglycaemia increased the odds of inpatient mortality and was associated with prolonged length of stay. Insulin therapy and non-White ethnicity were associated with developing inpatient hypoglycaemia. This finding is consistent with previous studies showing ethnic disparities in the management (including deintensification rate) and outcomes of people with diabetes.^18^, ^19^, ^20^ Our study showed for the first time an increased odds of inpatient hypoglycaemia in ethnic minorities with diabetes and frailty. It is, therefore, important to prevent inpatient hypoglycaemia with early medication review and early deintensification, especially in non-White populations who seem to be at higher risk. This could help to decrease morbidity and mortality as well as reduce the ethnic disparities in people with diabetes and frailty admitted to the hospital.

### Strengths and limitations

This study has several strengths. We included all patients discharged in the past year hence minimised the selection bias from the population. Our sample is also ethnically diverse which can be reflective of the general population in the United Kingdom. We also used a validated measurement of frailty which enabled us to include younger patients with frailty which was not done in previous studies. Our analyses also investigated several factors including age, CFS, background medications and co-morbidities which could also impact the measured outcomes.

One of the limitations of this study is the single-centredness making our deintensification rate not generalisable as this could be centre-specific. However, the rates of hypoglycaemia and its association with prolonged length of stay can be generalised as these are usually unaffected by the setting. Lastly, we also did not consider factors such as uncontrolled hyperglycaemia during admission and patient preference as these factors could potentially impact deintensification rates.

## Conclusion

Our study shows suboptimal assessment of HbA1c and a low rate of deintensification in people with diabetes and moderate/severe frailty including in patients with a high risk of falls and hypoglycaemia. We also identified factors associated with inpatient outcomes, most importantly, inpatient hypoglycaemia, further highlighting the importance of hypoglycaemia prevention and early inpatient deintensification. Finally, an evidenced-based stratification tool to identify patients at the highest risk of hypoglycaemia and overtreatment is urgently needed to improve patient care and decrease inpatient morbidity and mortality.

## Declaration of competing interest

SS reports personal fees from Amgen, AstraZeneca, NAPP, Lilly, Merck Sharp & Dohme, Novartis, Novo Nordisk, Roche, Sanofi-Aventis, Abbott and Boehringer Ingelheim. Additionally, SS reports grants from AstraZeneca, Sanofi-Aventis, Servier, and Janssen.

E.M., M.F, H.L., A.T., T.F.Y., K.T., H.T., F.A., M.D. and K.H. declare no conflict of interest.

## Appendix Supplementary materials

Image, application 1

## References

[1] Hudson MS, Palermo NE (2018). Principles and Practice of Geriatric Surgery: Third Edition: With 261 Figures and 155 Tables.

[2] Sinclair AJ, Abdelhafiz AH, Forbes A, Munshi M (2019). Evidence-based diabetes care for older people with Type 2 diabetes: a critical review. Diabet Med.

[3] Strain WD, Down S, Brown P, Puttanna A, Sinclair A (2021). Diabetes and frailty: an expert consensus statement on the management of older adults with Type 2 diabetes. Diabetes Therapy.

[4] Sternberg SA, Schwartz AW, Karunananthan S, Bergman H, Mark Clarfield A (2011). The identification of frailty: a systematic literature review. J Am Geriatr Soc.

[5] Gordon EH, Hubbard RE (2022). Frailty: understanding the difference between age and ageing. Age Ageing.

[6] Khunti K, Davies MJ (2017). Clinical inertia-Time to reappraise the terminology?. Prim Care Diabetes.

[7] Seidu S, Seewoodharry M, Khunti K (2021). De-intensification in older people with type 2 diabetes: why, when and for whom?. Lancet Healthy Longev.

[8] Lederle LI, Steinman MA, Jing B, Nguyen B, Lee SJ (2022). Glycemic treatment deintensification practices in nursing home residents with type 2 diabetes. J Am Geriatr Soc.

[9] Seidu S, Than T, Kar D, Lamba A, Brown P, Zafar A (2018). Therapeutic inertia amongst general practitioners with interest in diabetes. Prim Care Diabetes.

[10] Alexopoulos AS, Kahkoska AR, Pate V, Bradley MC, Niznik J, Thorpe C (2021). Deintensification of treatment with sulfonylurea and insulin after severe hypoglycemia among older adults with diabetes. JAMA Netw Open.

[11] JBDS 15 inpatient care of the frail older adult with diabetes | The Association of British Clinical Diabetologists. 2023 [cited 2023 Sep 15]. Available from: https://abcd.care/resource/current/jbds-15-inpatient-care-frail-older-adult-diabetes.

[12] Pasquel FJ, Powell W, Peng L, Johnson TM, Sadeghi-Yarandi S, Newton C (2015). A randomized controlled trial comparing treatment with oral agents and basal insulin in elderly patients with type 2 diabetes in long-term care facilities. BMJ Open Diabetes Res Care.

[13] Christiaens A, Henrard S, Boland B, Sinclair AJ (2023). Overtreatment of older people with type 2 diabetes-a high impact frequent occurrence in need of a new definition. Diabet Med.

[14] Christy AL, Manjrekar PA, Babu RP, Hegde A, Rukmini MS (2014). Influence of iron deficiency anemia on hemoglobin A1C levels in diabetic individuals with controlled plasma glucose levels. Iran Biomed J.

[15] Seidu S, Kunutsor SK, Topsever P, Hambling CE, Cos FX, Khunti K (2019). Deintensification in older patients with type 2 diabetes: a systematic review of approaches, rates and outcomes. Diabetes Obes Metab.

[16] Abdelhafiz AH, Rodríguez-Mañas L, Morley JE, Sinclair AJ (2015). Hypoglycemia in older people - a less well recognized risk factor for frailty. Aging Dis.

[17] Bonds DE, Miller ME, Dudl J, Feinglos M, Ismail-Beigi F, Malozowski S (2012). Severe hypoglycemia symptoms, antecedent behaviors, immediate consequences and association with glycemia medication usage: secondary analysis of the ACCORD clinical trial data. BMC Endocr Disord.

[18] Mathur R, Farmer RE, Eastwood SV, Chaturvedi N, Douglas I, Smeeth L (2020). Ethnic disparities in initiation and intensification of diabetes treatment in adults with type 2 diabetes in the UK, 1990–2017: a cohort study. PLoS Med.

[19] Karter AJ, Laiteerapong N, Chin MH, Moffet HH, Parker MM, Sudore R (2015). Ethnic differences in geriatric conditions and diabetes complications among older, insured adults with diabetes: the diabetes & aging study. J Aging Health.

[20] Malawana M, Hutchings A, Mathur R, Robson J (2018). Ethnic variations in the risk of hypoglycaemia among people with Type 2 diabetes prescribed insulins and/or sulfonylureas: a historical cohort study using general practice-recorded data. Diabet Med.

[21] Fried LP, Tangen CM, Walston J, Newman AB, Hirsch C, Gottdiener J (2001). Frailty in older adults: evidence for a phenotype. J Gerontol: Ser A.

[22] Morley JE, Malmstrom TK, Miller DK (2012). A simple frailty questionnaire (frail) predicts outcomes in middle aged African Americans. J Nutr Health Aging.

[23] Raîche M, Hébert R, Dubois MF (2008). PRISMA-7: a case-finding tool to identify older adults with moderate to severe disabilities. Arch Gerontol Geriatr.

