# Supplementary material for: Tertiary centre study highlights low inpatient deintensification and risks associated with adverse outcomes in frail people with diabetes
Source: Clin Med (Lond). 2024 Feb 20;24(2):100029. doi: 10.1016/j.clinme.2024.100029 (PMC11091443; doi:10.1016/j.clinme.2024.100029)
Supplement: Supplementary file 1 [file mmc1.docx]

Appendix 1. Baseline characteristics of inpatients with diabetes and moderate/severe frailty who are not overtreated stratified by HbA1c levels.

|  | Total | Optimally treated  (HbA1c 7-8.5% or  53-69mmol/mol)  n=187 | Not optimally treated (HbA1c > 8.5% or 69mmol/mol)  n=127 | p-value |
| --- | --- | --- | --- | --- |
| Age [median (IQR)] | 79 (71-85) | 79 (71-85) | 78 (69-84) | 0.142 |
| Female, n (%) | 156, 49.7% | 78, 41.7% | 78, 61.4% | 0.001 |
| BMI [median (IQR)] | 26.45 (22.82-31.67) | 26.35 (23.02-31.48) | 26.79 (22.64-32.29) | 0.851 |
| **Ethnicity**, n (%) White Non-white | 241, 76.8% 73, 23.2% | 147, 78.6%  40, 21.4% | 94, 74.0%  33, 26.0% | 0.344 |
| **CFS**, n (%) Moderate frailty Severe frailty  Very severe frailty Terminally ill | 218, 69.4%  76, 24.2%  17, 5.4%  3, 1.0% | 131, 70.1%  39, 20.9%  15, 8.0%  2, 1.1% | 87, 68.5%  37, 29.1%  2, 1.6%  1, 0.8% | 0.026 |
| **CFS,** median (IQR) | 6 (6-7) | 6 (6-7) | 6 (6-7) |  |
| **Co-morbidities**  Retinopathy CKD/Nephropathy PAD/Amputation Neuropathy Heart failure IHD Cerebrovascular event Hypertension Dementia Dyslipidaemia | 37 (11.8%)  176, 56.1%  22, 7.0%  14, 4.5%  75, 23.9%  93, 29.6%  66, 21.0%  205, 65.3%  83, 26.4%  69, 22.0% | 20, 10.7%  100, 53.5%  12, 6.4%  8, 4.3%  45, 24.1%  62, 33.2%  34, 18.2%  131, 64.7%  48, 25.7%  48, 25.7% | 17, 13.4%  76, 59.8%  10, 7.9%  6, 4.7%  30, 23.6%  31, 24.4%  32, 25.2%  84, 66.1%  35, 27.6%  21, 16.5% | 0.468  0.265  0.620  0.851  0.928  0.096  0.134  0.793  0.709  0.055 |
| **Glucose-lowering agents** Metformin Sulphonylureas Insulin GLP1ra DPP4i SGLT2i | 133, 42.4%  40, 12.7%  174, 55.4%  8, 2.5%  70, 22.3%  22, 7.0% | 88, 47.1%  23, 12.3%  77, 41.2%  2, 1.1%  40, 21.4%  11, 5.9% | 45, 35.4%  17, 13.4%  76.4%  6, 4.7%  30, 23.6%  11, 8.7% | 0.041  0.777  <0.001  0.044  0.641  0.344 |
| At least one glucose-lowering agent | 277, 88.2% | 153, 81.8% | 124, 97.6% | <0.001 |
| **Sulphonylureas or Insulin** None At least one Both | 111, 35.4%  192, 61.1%  11, 3.5% | 90, 48.1%  94, 50.3%  3, 1.6% | 21, 16.5%  98, 77.2%  8, 6.3% | <0.001 |
| Admission HbA1c%  [median (IQR)] | 8.3 (7.5-9.5) | 7.7 (7.3-7.1) | 10 (9.0-11.1) | <0.001 |
| Admission HbA1c, mmol/mol  [median (IQR)] | 67 (58-80) | 60 (56-64) | 86 (76-98) | <0.001 |
| If on metformin, was vitamin B12 checked in the last 1 year, n (%) | 71/133, 53.4% | 45, 70.3% | 26, 57.8% | 0.096 |
| Admission due to fall, n (%) | 88/298, 29.5% | 54, 30.7% | 34, 27.9% | 0.601 |
| Inpatient hypoglycaemia, n (%) | 70/302, 23.2% | 33, 18.5% | 37, 29.8% | 0.022 |
| Inpatient mortality, n (%) | 19/314, 6.1% | 14, 7.5% | 5, 3.9% | 0.195 |
| Length of stay [median (IQR)] | 7 (3-17) | 6 (3-15.5) | 10 (3-18) | 0.092 |
| Deintensification, n (%) | 73/296, 24.7% | 39, 22.5% | 34, 27.6% | 0.316 |
| Readmission in one month, n (%) | 69/300, 23.0% | 39, 22.2% | 30, 24.2% | 0.680 |
| Readmission in one month due to fall, n (%) | 11/70, 15.7% | 6, 15.0% | 5, 16.7% | 0.850 |

IQR = interquartile range; BMI = body mass index; HbA1c = glycated haemoglobin A1c; CFS: clinical frailty score; Optimally controlled = HbA1c 7-8.5% (53-69 mmol/mol); Not optimally controlled = HbA1c >8.5% (>69 mmol/mol).

Appendix 2. Baseline characteristics of inpatients with diabetes and moderate/severe frailty stratified by sex

|  | Total | Female, n=345 | Male, n=320 | p-value |
| --- | --- | --- | --- | --- |
| Age [median (IQR)] | 79 (71-86) | 81 (71-86) | 78 (71-85) | 0.056 |
| Overtreated, n (%) | 335, 50.4% | 180, 52.2% | 155, 48.4% | 0.336 |
| BMI [median (IQR)] | 25.79 (22.09-30.81) | 305, 25.39 (22.28-29.63) | 331, 26.35 (21.65-31.64) | 0.112 |
| **Ethnicity**, n (%) White Non-white | 504, 75.8% 161, 24.2% | 253, 73.3% 92, 26.7% | 251, 78.4% 69, 21.6% | 0.125 |
| **CFS**, n (%) Moderate frailty Severe frailty  Very severe frailty Terminally ill | 448/665, 67.4% 176/665, 26.5% 29/665, 4.4% 12/665, 1.8% | 236, 68.4% 86, 24.9% 18, 5.2% 5, 1.4% | 212, 66.3% 90, 28.1% 11, 3.4% 7, 2.2% | 0.482 |
| Admission HbA1c  [median (IQR)] | 6.9 (6.20-8.1) | 6.9 (6.2-8.3) | 7.0 (6.2-8.0) | 0.376 |
| If on metformin, was vitamin B12 checked in the last 1 year, n (%) | 136/247, 55.1% | 64, 56.6% | 72, 53.7% | 0.647 |
| Admission due to fall, n (%) | 170/628, 27.1% | 93, 28.6% | 77, 25.4% | 0.367 |
| Inpatient hypoglycaemia, n (%) | 116/637, 18.2% | 60, 18.1% | 56, 18.3% | 0.955 |
| Inpatient mortality, n (%) | 49/665, 7.4% | 27, 7.8% | 22, 6.9% | 0.639 |
| Length of stay [median (IQR)] | 651, 8 (3-16) | 8 (4-17) | 8 (3-15) | 0.742 |
| Deintensification, n (%) | 119/624, 19.1% | 58, 17.9% | 61, 20.3% | 0.440 |
| Readmission in one month, n (%) | 153/634, 24.1% | 73, 21.9% | 80, 26.7% | 0.158 |
| Readmission in one month due to fall, n (%) | 29/153, 19.0% | 15, 20.5% | 14, 17.5% | 0.631 |

IQR = interquartile range; BMI = body mass index; HbA1c = glycated haemoglobin A1c; CFS: clinical frailty score; Overtreated = HbA1c <7.0% + at least one glucose-lowering medication

Appendix 3. Baseline characteristics of inpatients with diabetes and moderate/severe frailty stratified by ethnicity

|  | Total, 665 (100%) | White, 504 (75.8%) | Non-white, 161 (24.2%) | p-value |
| --- | --- | --- | --- | --- |
| Age [median (IQR)] | 79 (71-86) | 79 (71-86) | 81 (71-85) | 0.576 |
| Female, n (%) | 345, 51.9% | 253, 50.2% | 92, 57.1% | 0.125 |
| Overtreated, n (%) | 335, 50.4% | 249, 49.4% | 86, 53.4% | 0.375 |
| BMI [median (IQR)] | 25.79 (22.1-30.8) | 26.4 (22.6-31.6) | 24.4 (20.3-29.0) | 0.001 |
| **CFS**, n (%) Moderate frailty Severe frailty  Very severe frailty Terminally ill | 448/665, 67.4% 176/665, 26.5% 29/665, 4.4% 12/665, 1.8% | 348, 69.0% 127, 25.2% 21, 4.2% 8, 1.6% | 100, 62.1% 49, 30.4% 8, 5.0% 4, 2.5% | 0.360 |
| At least one glucose-lowering agent, n (%) | 467, 70.2% | 353, 70.0% | 114, 70.8% | 0.853 |
| **Sulphonylureas or Insulin**, n (%) None At least one Both | 397, 59.7% 254, 38.2% 14, 2.1% | 302, 59.9% 192, 38.1% 10, 2.0% | 95, 59.0% 62, 38.5% 4, 2.5% | 0.819 |
| Admission HbA1c  [median (IQR)] | 6.9 (6.2-8.1) | 7.0 (6.2-8.1) | 6.8 (6.3-8.1) | 0.426 |
| If on metformin, was vitamin B12 checked in the last 1 year, n (%) | 136/247, 55.1% | 100, 52.9% | 36, 62.1% | 0.220 |
| Admission due to fall, n (%) | 170/628, 27.1% | 137, 28.4% | 33, 22.6% | 0.166 |
| Inpatient hypoglycaemia, n (%) | 116/637, 18.2% | 82, 16.7% | 34, 23.4% | 0.063 |
| Inpatient mortality, n (%) | 49/665, 7.4% | 30, 6.0% | 19, 11.8% | 0.013 |
| Length of stay  [median (IQR)] | 651, 8 (3-16) | 8.0 (3-16) | 8 (3-14) | 0.654 |
| Deintensification, n (%) | 119/624, 19.1% | 91, 19.0% | 28, 19.4% | 0.896 |
| Readmission in one month, n (%) | 153/634, 24.1% | 121, 24.8% | 32, 21.9% | 0.476 |
| Readmission in one month due to fall, n (%) | 29/153, 19.0% | 21, 17.6% | 8, 23.5% | 0.440 |

IQR = interquartile range; BMI = body mass index; HbA1c = glycated haemoglobin A1c; CFS: clinical frailty score; Overtreated = HbA1c <7.0% + at least one glucose-lowering medication

Appendix 4. Baseline characteristics of inpatients with diabetes and moderate/severe frailty stratified by age group.

|  | Total | <65 years old | ≥65 years old | p-value |
| --- | --- | --- | --- | --- |
| Female, n (%) | 345, 51.9% | 41, 53.9% | 304, 51.6% | 0.701 |
| Overtreated | 335, 50.4% | 38, 50.0% | 297, 50.4% | 0.944 |
| BMI [median (IQR)] | 25.79 (22.09-30.81) | 28.0 (10.9-34.7) | 25.5 (21.8-30.3) | <0.001 |
| **Ethnicity**, n (%)  White Non-white | 504, 75.8% 161, 24.2% | 67, 88.2% 9, 11.8% | 437, 74.2% 152, 25.8% | 0.007 |
| **CFS**, n (%) Moderate frailty Severe frailty  Very severe frailty Terminally ill | 448/665, 67.4% 176/665, 26.5% 29/665, 4.4% 12/665, 1.8% | 53, 69.7% 13, 17.1% 7, 9.2% 3, 3.9% | 395, 67.1% 163, 27.7% 22, 3.7% 9, 1.5% | 0.021 |
| **Co-morbidities**, n (%)  Retinopathy CKD/Nephropathy PAD/Amputation Neuropathy Heart failure IHD Cerebrovascular event Hypertension Dementia Dyslipidaemia | 62, 9.3% 362, 54.4% 40, 6.0% 26, 3.9% 167, 25.1% 177, 26.6% 135, 20.3% 448, 67.4% 159, 23.9% 128, 19.2% | 8, 10.5% 22, 28.9% 2, 2.6% 3, 3.9% 7, 9.2% 6, 7.9% 17, 22.4% 36, 47.4% 4, 5.3% 13, 17.1% | 54, 9.2% 340, 57.7% 38, 6.5% 23, 3.9% 160, 27.2% 171, 29.0% 118, 20.0% 412, 69.9% 155, 26.3% 115, 19.5% | 0.702 <0.001 0.187 0.986 0.001 0.000 0.634 <0.001 <0.001 0.615 |
| Admission HbA1c  [median (IQR)] | 6.9 (6.20-8.1) | 7.0 (5.9-8.6) | 6.9 (6.3-8.1) | 0.958 |
| If on metformin, was vitamin B12 checked in the last 1 year, n (%) | 136/247, 55.1% | 16, 51.6% | 120, 55.6% | 0.680 |
| Admission due to fall, n (%) | 170/628, 27.1% | 8, 10.7% | 162, 29.3% | 0.001 |
| Inpatient hypoglycaemia, n (%) | 116/637, 18.2% | 14, 19.7% | 105, 19.0% | 0.773 |
| Inpatient mortality, n (%) | 49/665, 7.4% | 5, 6.6% | 44, 7.5% | 0.780 |
| Length of stay [median (IQR)] | 651, 8 (3-16) | 5 (2-11) | 8 (4-16) | 0.040 |
| Deintensification, n (%) | 119/624, 19.1% | 14, 19.7% | 105, 19.0% | 0.883 |
| Readmission in one month, n (%) | 153/634, 24.1% | 22, 30.6% | 131, 23.3% | 0.176 |
| Readmission in one month due to fall, n (%) | 29/153, 19.0% | 0, 0.0% | 29, 22.0% | 0.014 |

IQR = interquartile range; BMI = body mass index; HbA1c = glycated haemoglobin A1c; CFS: clinical frailty score; Overtreated = HbA1c <7.0% + at least one glucose-lowering medication

Appendix 5. Unadjusted and adjusted odds ratio for developing severe inpatient hypoglycaemia in people with diabetes and moderate/severe frailty

| Variable | OR (95% CI) | p value | aOR(95% CI) | p value |
| --- | --- | --- | --- | --- |
| Age > 65 | 0.68 (0.29 - 1.59) | 0.377 | 1.01 (0.36 - 2.86) | 0.984 |
| Female sex | 0.75 (0.39 - 1.42) | 0.37 | 0.50 (0.23 - 1.09) | 0.083 |
| Ethnic minority | 1.55 (0.78 - 3.08) | 0.21 | 1.85 (0.82 - 4.18) | 0.141 |
| Retinopathy | 0.79 (0.23 - 2.65) | 0.706 | 0.54 (0.14 - 2.06) | 0.364 |
| Chronic kidney disease/Nephropathy | 1.01 (0.53 - 1.91) | 0.984 | 1.32 (0.59 - 2.94) | 0.501 |
| Peripheral arterial disease/Amputation | 0.39 (0.05 - 2.93) | 0.361 | 0.19 (0.02 - 1.69) | 0.136 |
| Neuropathy | 0.63 (0.08 - 4.75) | 0.651 | 0.87 (0.09 - 7.99) | 0.9 |
| Heart Failure | 1.02 (0.48 - 2.13) | 0.965 | 0.65 (0.25 - 1.66) | 0.368 |
| Ischaemic heart disease | 0.94 (0.45 - 1.96) | 0.864 | 0.92 (0.38 - 2.22) | 0.858 |
| Cerebrovascular events | 0.84 (0.36 - 1.95) | 0.69 | 0.71 (0.27 - 1.85) | 0.483 |
| Hypertension | 1.06 (0.53 - 2.14) | 0.863 | 1.20 (0.52 - 2.76) | 0.675 |
| Dementia | 0.55 (0.23 - 1.35) | 0.194 | 0.47 (0.17 - 1.27) | 0.138 |
| Dyslipidaemia | 0.59 (0.23 - 1.55) | 0.288 | 0.61 (0.21 - 1.73) | 0.35 |
| Metformin | 0.40 (0.18 - 0.89) | 0.025 | 0.32 (0.12 - 0.82) | 0.018 |
| Sulphonylureas | 1.13 (0.39 - 3.31) | 0.817 | 2.21 (0.65 - 7.48) | 0.204 |
| Insulin | 3.76 (1.91 - 7.39) | < 0.01 | 3.39 (1.44 - 8.00) | 0.005 |
| DPP4i | 0.97 (0.42 - 2.26) | 0.952 | 0.62 (0.24 - 1.64) | 0.336 |
| SGLT2i | 1.29 (0.38 - 4.39) | 0.683 | 1.24 (0.32 - 4.79) | 0.758 |
| Admission HbA1c < 7% | 0.57 (0.30 - 1.10) | 0.097 | 0.84 (0.35 - 1.97) | 0.683 |
| Overtreated 1 | 0.91 (0.44 - 1.91) | 0.811 | 1.10 (0.47 - 2.61) | 0.823 |
| Overtreated 2 | 0.56 (0.27 - 1.18) | 0.127 | 0.63 (0.27 - 1.46) | 0.279 |
| Overtreated 3 | 0.73 (0.38 - 1.42) | 0.359 | 0.77 (0.35 - 1.67) | 0.505 |
| Overtreated 4 | 0.80 (0.42 - 1.52) | 0.496 | 0.81 (0.37 - 1.76) | 0.598 |
| Admission due to fall | 0.48 (0.20 - 1.16) | 0.102 | 0.55 (0.21 - 1.48) | 0.237 |

OR: odds ratio; aOR = adjusted for age, sex, ethnicity, CFS, background co-morbidities, background medications, admission HbA1c and admission due to fall; DPP4i = dipeptidyl peptidase 4 inhibitors; SGLT2i = Sodium-glucose co-transporter-2; Overtreated 1 = HbA1c <7.0% + at least one glucose-lowering medication; Overtreated 2 = HbA1c <7.5% + at least one glucose-lowering medication; Overtreated 3 = HbA1c <8.0% + at least one glucose-lowering medication; Overtreated 4 = HbA1c <8.5% + at least one glucose-lowering medication

Appendix 6. Unadjusted and adjusted odds ratio for inpatient deintensification in people with diabetes and moderate/severe frailty

| Variable | OR (95% CI) | p value | aOR(95% CI) | p value |
| --- | --- | --- | --- | --- |
| Age > 65 | 0.95 (0.53 - 1.71) | 0.867 | 0.83 (0.38 - 1.84) | 0.654 |
| Female sex | 0.85 (0.57 - 1.27) | 0.44 | 0.88 (0.54 - 1.45) | 0.622 |
| Ethnic minority | 1.03 (0.64 - 1.65) | 0.896 | 0.91 (0.51 - 1.63) | 0.749 |
| Retinopathy | 1.23 (0.64 - 2.36) | 0.533 | 0.79 (0.36 - 1.71) | 0.545 |
| Chronic kidney disease/Nephropathy | 1.27 (0.85 - 1.92) | 0.238 | 1.77 (1.03 - 3.03) | 0.018 |
| Peripheral arterial disease/Amputation | 1.31 (0.61 - 2.85) | 0.489 | 1.03 (0.39 - 2.74) | 0.95 |
| Neuropathy | 1.44 (0.56 - 3.71) | 0.447 | 1.34 (0.42 - 4.28) | 0.617 |
| Heart Failure | 0.87 (0.55 - 1.40) | 0.574 | 0.84 (0.46 - 1.51) | 0.55 |
| Ischaemic heart disease | 1.15 (0.74 - 1.79) | 0.528 | 0.95 (0.55 - 1.51) | 0.846 |
| Cerebrovascular events | 0.88 (0.53 - 1.47) | 0.628 | 0.84 (0.45 - 1.57) | 0.579 |
| Hypertension | 1.21 (0.78 - 1.87) | 0.404 | 1.39 (0.79 - 2.43) | 0.249 |
| Dementia | 1.19 (0.75 - 1.87) | 0.459 | 1.09 (0.63 - 1.89) | 0.758 |
| Dyslipidaemia | 1.27 (0.79 - 2.06) | 0.326 | 0.92 (0.50 - 1.69) | 0.787 |
| Metformin | 1.67 (1.11 - 2.50) | 0.013 | 2.22 (1.31 - 3.78) | 0.003 |
| Sulphonylureas | 2.94 (1.64 - 5.29) | < 0.01 | 4.23 (2.04 - 8.74) | <0.01 |
| Insulin | 3.16 (2.10 - 4.77) | < 0.01 | 3.10 (1.70 - 5.66) | <0.01 |
| DPP4i | 0.99 (0.59 - 1.66) | 0.977 | 0.67 (0.35 - 1.25) | 0.21 |
| SGLT2i | 2.04 (1.00 - 4.18) | 0.05 | 3.06 (1.27 - 7.4) | 0.014 |
| Admission HbA1c < 7% | 0.54 (0.36 - 0.82) | 0.003 | 1.11 (0.64 - 1.92) | 0.704 |
| Overtreated 1 | 1.53 (1.00 - 2.35) | 0.051 | 1.65 (0.97 - 2.80) | 0.066 |
| Overtreated 2 | 1.31 (0.87 - 1.97) | 0.196 | 1.15 (0.69 - 1.94) | 0.576 |
| Overtreated 3 | 1.75 (1.17 - 2.62) | 0.006 | 1.41 (0.85 - 2.34) | 0.183 |
| Overtreated 4 | 1.95 (1.29 - 2.95) | 0.001 | 1.51 (0.89 - 2.55) | 0.125 |
| Admission due to fall | 1.33 (0.86 - 2.07) | 0.199 | 1.58 (0.93 - 2.71) | 0.093 |
| Inpatient hypoglycaemia | 6.22 (3.96 - 9.78) | < 0.01 | 4.97 (2.89 - 8.57) | <0.01 |
| Inpatient severe hypoglycaemia | 3.91 (2.02 - 7.56) | < 0.01 | 2.71 (1.23 - 5.94) | <0.01 |
|  |  |  |  |  |
| OR: odds ratio; aOR = adjusted for age, sex, ethnicity, CFS, background co-morbidities, background medications, admission HbA1c and admission due to fall; DPP4i = dipeptidyl peptidase 4 inhibitors; SGLT2i = Sodium-glucose co-transporter-2; Overtreated 1 = HbA1c <7.0% + at least one glucose-lowering medication; Overtreated 2 = HbA1c <7.5% + at least one glucose-lowering medication; Overtreated 3 = HbA1c <8.0% + at least one glucose-lowering medication; Overtreated 4 = HbA1c <8.5% + at least one glucose-lowering medication | | | | |

Appendix 7. Linear regression and factors associated with length of stay in people with diabetes and moderate/severe frailty

| Variable | Regression co-efficient | 95% CI | p value |
| --- | --- | --- | --- |
| Age | -0.02 | -0.13 - 0.08 | 0.649 |
| Sex | 1.68 | -0.48 3.85 | 0.127 |
| Ethnicity | -0.41 | -2.96 - 2.14 | 0.754 |
| CFS | -1.15 | -2.81 - 0.51 | 0.175 |
| BMI | 0.13 | -0.01 - 0.27 | 0.069 |
| Admission HbA1c | 0.15 | -0.42 - 0.72 | 0.615 |
| Retinopathy | -0.32 | -4.00 - 3.36 | 0.864 |
| Chronic kidney disease/Nephropathy | -0.34 | -2.50 - 1.82 | 0.76 |
| Peripheral arterial disease/Amputation | -1.14 | -5.60 - 3.33 | 0.617 |
| Neuropathy | 2.6 | -2.87 - 8.06 | 0.352 |
| Heart Failure | 1.25 | -1.22 - 3.72 | 0.323 |
| Ischaemic heart disease | 0.38 | -2.04 - 2.80 | 0.759 |
| Cerebrovascular events | 0.35 | -2.30 - 3.01 | 0.794 |
| Hypertension | 0.26 | -2.04 - 2.56 | 0.822 |
| Dementia | -1.87 | -4.37 - 0.64 | 0.144 |
| Dyslipidaemia | -0.59 | -3.31 - 2.12 | 0.667 |
| Metformin | -0.03 | -2.29 - 2.22 | 0.976 |
| Sulphonylureas | -1.27 | -5.08 - 2.55 | 0.516 |
| Insulin | 3.14 | 0.85 - 5.43 | 0.007 |
| DPP4i | 2.22 | -0.6 - 5.04 | 0.122 |
| SGLT2i | 0.82 | -3.71 - 5.35 | 0.724 |
| Admission HbA1c | 0.15 | -0.42 - 0.72 | 0.615 |
| Admission due to fall | 0.43 | -2.00 - 2.85 | 0.729 |
| Inpatient hypoglycaemia | 6.55 | 3.77 - 9.32 | <0.01 |
| Inpatient severe hypoglycaemia | 8.35 | 3.88 - 12.8 | <0.01 |

CFS = clinical frailty score; BMI = body mass index; HbA1c = glycated haemoglobin A1c; DPP4i = dipeptidyl peptidase 4 inhibitors; SGLT2i = Sodium-glucose co-transporter-2

Appendix 8. Unadjusted and adjusted odds ratio for inpatient mortality in people with diabetes and moderate/severe frailty

| Variable | OR (95% CI) | p value | aOR(95% CI) | p value |
| --- | --- | --- | --- | --- |
| Age > 65 | 1.33 (0.51 - 3.46) | 0.556 | 5.52 (0.50 - 60.5) | 0.873 |
| Female sex | 1.15 (0.64 - 2.06) | 0.639 | 0.82 (0.26 - 2.65) | 0.745 |
| Ethnic minority | 2.11 (1.16 - 3.87) | 0.015 | 1.41 (0.37 - 5.39) | 0.612 |
| Retinopathy | 1.22 (0.46 - 3.22) | 0.686 | 3.31 (0.72 - 15.3) | 0.125 |
| Chronic kidney disease/Nephropathy | 0.81 (0.45 - 1.48) | 0.494 | 0.66 (0.20 - 2.14) | 0.492 |
| Neuropathy | 1.14 (0.26 - 5.00) | 0.859 | 2.54 (0.33 - 19.8) | 0.374 |
| Heart Failure | 0.95 (0.47 - 1.92) | 0.886 | 0.71 (0.18 - 2.78) | 0.621 |
| Ischaemic heart disease | 0.66 (0.31 - 1.41) | 0.288 | 0.65 (0.16 - 2.60) | 0.547 |
| Cerebrovascular events | 0.83 (0.38 - 1.83) | 0.645 | 0.58 (0.11 - 3.07) | 0.522 |
| Hypertension | 0.62 (0.34 - 1.15) | 0.131 | 0.15 (0.04 - 0.50) | 0.002 |
| Dementia | 0.56 (0.24 - 1.28) | 0.169 | 0.27 (0.05 - 1.46) | 0.129 |
| Dyslipidaemia | 0.75 (0.33 - 1.72) | 0.498 | 1.01 (0.20 - 5.07) | 0.994 |
| Metformin | 1.34 (0.70 - 2.61) | 0.375 | 0.62 (0.17 - 2.32) | 0.482 |
| Sulphonylureas | 1.17 (0.40 - 3.41) | 0.774 | 1.32 (0.14 - 12.9) | 0.81 |
| Insulin | 1.14 (0.58 - 2.24) | 0.714 | 1.93 (0.47 - 7.94) | 0.362 |
| DPP4i | 0.82 (0.34 - 2.03) | 0.68 | 1.02 (0.24 - 4.33) | 0.977 |
| SGLT2i | 1.37 (0.40 - 4.67) | 0.614 | 0.81 (0.08 - 8.82) | 0.865 |
| Admission HbA1c < 7% | 1.47 (0.81 - 2.65) | 0.202 | 1.89 (0.51 - 7.01) | 0.344 |
| Overtreated 1 | 1.00 (0.51 - 1.92) | 0.989 | 3.83 (1.06 - 13.8) | 0.04 |
| Overtreated 2 | 1.02 (0.56 - 1.86) | 0.959 | 2.50 (0.69 - 9.06) | 0.162 |
| Overtreated 3 | 0.85 (0.47 - 1.53) | 0.589 | 1.30 (0.36 - 4.69) | 0.688 |
| Overtreated 4 | 0.73 (0.40 - 1.31) | 0.285 | 1.04 (0.28 - 3.86) | 0.955 |
| Admission due to fall | 0.75 (0.32 - 1.77) | 0.515 | 2.12 (0.56 - 8.08) | 0.267 |
| Inpatient hypoglycaemia | 2.23 (0.98 - 5.06) | 0.06 | 3.57 (1.02 - 12.4) | 0.046 |
| Inpatient severe hypoglycaemia | 1.02 (0.30 - 3.43) | 0.974 | 1.56 (0.31 - 7.99) | 0.593 |
|  |  |  |  |  |
| OR = odds ratio; aOR = adjusted for age, sex, ethnicity, CFS, background co-morbidities, background medications, admission HbA1c, inpatient hypoglycaemia and severe hypoglycaemia and admission due to fall; Overtreated 1 = HbA1c <7.0% + at least one glucose-lowering medication; Overtreated 2 = HbA1c <7.5% + at least one glucose-lowering medication; Overtreated 3 = HbA1c <8.0% + at least one glucose-lowering medication; Overtreated 4 = HbA1c <8.5% + at least one glucose-lowering medication | | | | |

Appendix 9. Unadjusted and adjusted odds ratio for one-month readmission in people with diabetes and moderate/severe frailty

| Variable | OR (95% CI) | p value | aOR(95% CI) | p value |
| --- | --- | --- | --- | --- |
| Age > 65 | 0.64 (0.39 - 1.07) | 0.088 | 0.46 (0.25 - 0.85) | 0.013 |
| Female sex | 0.77 (0.53 - 1.11) | 0.158 | 0.90 (0.59 - 1.36) | 0.603 |
| Ethnic minority | 0.85 (0.55 - 1.33) | 0.476 | 0.86 (0.52 - 1.43) | 0.571 |
| Retinopathy | 1.19 (0.65 - 2.19) | 0.568 | 1.17 (0.59 - 2.33) | 0.647 |
| Chronic kidney disease/Nephropathy | 0.97 (0.67 - 1.40) | 0.861 | 1.10 (0.71 - 1.70) | 0.681 |
| Peripheral arterial disease/Amputation | 1.86 (0.94 - 3.67) | 0.075 | 1.70 (0.78 - 3.72) | 0.182 |
| Neuropathy | 0.44 (0.12 - 1.49) | 0.187 | 0.43 (0.11 - 1.58) | 0.202 |
| Heart Failure | 0.98 (0.64 - 1.49) | 0.908 | 0.88 (0.53 - 1.45) | 0.613 |
| Ischaemic heart disease | 1.49 (1.00 - 2.21) | 0.051 | 1.67 (1.07 - 2.61) | 0.023 |
| Cerebrovascular events | 0.99 (0.63 - 1.55) | 0.964 | 0.99 (0.60 - 1.64) | 0.975 |
| Hypertension | 1.11 (0.75 - 1.65) | 0.596 | 1.35 (0.86 - 2.13) | 0.191 |
| Dementia | 0.76 (0.49 - 1.17) | 0.211 | 0.74 (0.45 - 1.22) | 0.24 |
| Dyslipidaemia | 0.77 (0.47 - 1.24) | 0.278 | 0.73 (0.43 - 1.24) | 0.242 |
| Metformin | 0.91 (0.62 - 1.34) | 0.636 | 1.00 (0.64 - 1.55) | 0.994 |
| Sulphonylureas | 0.74 (0.37 - 1.46) | 0.383 | 0.96 (0.45 - 2.06) | 0.928 |
| Insulin | 1.20 (0.82 - 1.75) | 0.356 | 1.11 (0.65 - 1.88) | 0.706 |
| DPP4i | 1.07 (0.67 - 1.70) | 0.792 | 1.11 (0.67 - 1.86) | 0.678 |
| SGLT2i | 1.33 (0.64 - 2.75) | 0.448 | 1.47 (0.66 - 3.30) | 0.35 |
| Admission HbA1c < 7% | 1.01 (0.70 - 1.45) | 0.962 | 1.11 (0.70 - 1.76) | 0.129 |
| Overtreated 1 | 1.09 (0.72 - 1.64) | 0.682 | 1.13 (0.70 - 1.80) | 0.618 |
| Overtreated 2 | 1.27 (0.87 - 1.85) | 0.211 | 1.27 (0.81 - 2.01) | 0.296 |
| Overtreated 3 | 1.28 (0.89 - 1.85) | 0.181 | 1.29 (0.83 - 2.02) | 0.261 |
| Overtreated 4 | 1.28 (0.89 - 1.85) | 0.18 | 1.39 (0.88 - 2.20) | 0.124 |
| Admission due to fall | 1.12 (0.75 - 1.70) | 0.557 | 1.18 (0.90 - 1.15) | 0.481 |
| Inpatient hypoglycaemia | 0.95 (0.59 - 1.54) | 0.849 | 0.90 (0.46 - 1.77) | 0.76 |
| Inpatient severe hypoglycaemia | 1.05 (0.50 - 2.20) | 0.895 | 0.92 (0.34 - 2.47) | 0.866 |
| Deintensification | 0.93 (0.58 - 1.49) | 0.774 | 0.86 (0.50 - 1.51) | 0.607 |
|  |  |  |  |  |
| OR = odds ratio; aOR = adjusted for age, sex, ethnicity, CFS, background co-morbidities, background medications, admission HbA1c, inpatient hypoglycaemia and severe hypoglycaemia, inpatient deintensification and admission due to fall; Overtreated 1 = HbA1c <7.0% + at least one glucose-lowering medication; Overtreated 2 = HbA1c <7.5% + at least one glucose-lowering medication; Overtreated 3 = HbA1c <8.0% + at least one glucose-lowering medication; Overtreated 4 = HbA1c <8.5% + at least one glucose-lowering medication | | | | |

Appendix 10. Unadjusted and adjusted odds ratio for one-month readmission with fall in people with diabetes and moderate/severe frailty

| Variable | OR (95% CI) | p value | aOR(95% CI) | p value |
| --- | --- | --- | --- | --- |
| Age > 65 | 6.72 (0.87 - 51.9) | 0.068 | 7.57 (0.47 - 120.9) | 0.152 |
| Female sex | 1.22 (0.54 - 2.74) | 0.631 | 0.90 (0.26 - 3.23) | 0.883 |
| Ethnic minority | 1.44 (0.57 - 3.61) | 0.442 | 1.54 (0.42 - 5.73) | 0.517 |
| Retinopathy | 0.29 (0.04 - 2.31) | 0.243 | 0.34 (0.03 - 3.90) | 0.386 |
| Chronic kidney disease/Nephropathy | 1.13 (0.50 - 2.60) | 0.765 | 0.47 (0.13 - 1.66) | 0.24 |
| Peripheral arterial disease/Amputation | 0.78 (0.16 - 3.75) | 0.76 | 0.79 (0.08 - 7.86) | 0.838 |
| Heart Failure | 1.90 (0.79 - 4.59) | 0.151 | 2.07 (0.55 - 7.77) | 0.28 |
| Ischaemic heart disease | 1.53 (0.67 - 3.53) | 0.309 | 1.52 (0.48 - 4.90) | 0.478 |
| Cerebrovascular events | 0.79 (0.27 - 2.26) | 0.654 | 0.30 (0.06 - 1.54) | 0.149 |
| Hypertension | 0.93 (0.39 - 2.25) | 0.877 | 0.53 (0.14 - 2.08) | 0.366 |
| Dementia | 1.39 (0.53 - 3.64) | 0.504 | 1.19 (0.28 - 5.12) | 0.816 |
| Dyslipidaemia | 1.61 (0.57 - 4.51) | 0.368 | 3.64 (0.89 - 14.9) | 0.073 |
| Metformin | 0.41 (0.16 - 1.09) | 0.074 | 0.22 (0.05 - 0.92) | 0.038 |
| Sulphonylureas | 0.84 (0.17 - 4.08) | 0.833 | 0.83 (0.09 - 7.34) | 0.864 |
| Insulin | 0.50 (0.20 - 1.27) | 0.146 | 0.63 (0.13 - 3.00) | 0.559 |
| DPP4i | 1.77 (0.69 - 4.50) | 0.233 | 2.28 (0.62 - 8.34) | 0.212 |
| SGLT2i | 0.41 (0.05 - 3.31) | 0.401 | 0.42 (0.04 - 4.79) | 0.486 |
| Admission HbA1c < 7% | 1.56 (0.69 - 3.53) | 0.287 | 2.18 (0.56 - 8.43) | 0.474 |
| Overtreated 1 | 0.81 (0.32 - 2.06) | 0.657 | 2.46 (0.57 - 10.6) | 0.227 |
| Overtreated 2 | 0.58 (0.24 - 1.38) | 0.221 | 0.58 (0.14 - 2.38) | 0.452 |
| Overtreated 3 | 0.81 (0.36 - 1.83) | 0.616 | 1.19 (0.29 - 2.87) | 0.809 |
| Overtreated 4 | 0.61 (0.27 - 1.37) | 0.228 | 0.68 (0.17 - 2.73) | 0.585 |
| Admission due to fall | 3.73 (1.60 - 8.68) | 0.002 | 4.02 (1.04 - 15.5) | 0.044 |
| Inpatient hypoglycaemia | 0.66 (0.21 - 2.08) | 0.477 | 0.54 (0.07 - 3.94) | 0.541 |
| Inpatient severe hypoglycaemia | 0.41 (0.05 - 3.31) | 0.401 | 0.75 (0.04 - 13.7) | 0.844 |
| Deintensification | 0.67 (0.21 -2.10) | 0.488 | 1.62 (0.23 - 11.4) | 0.629 |
|  |  |  |  |  |
| OR = odds ratio; aOR = adjusted for age, sex, ethnicity, CFS, background co-morbidities, background medications, admission HbA1c, inpatient hypoglycaemia and severe hypoglycaemia, inpatient deintensification and admission due to fall; Overtreated 1 = HbA1c <7.0% + at least one glucose-lowering medication; Overtreated 2 = HbA1c <7.5% + at least one glucose-lowering medication; Overtreated 3 = HbA1c <8.0% + at least one glucose-lowering medication; Overtreated 4 = HbA1c <8.5% + at least one glucose-lowering medication | | | | |
